# Healthcare costs due to diplopia in Korea: analyses using the public data of Health Insurance Review and Assessment Service

**DOI:** 10.3389/fneur.2026.1922434

**Published:** 2026-08-13

**Authors:** Hyo-Jung Kim, Jae-Ryun Lee, Hyejin Lee, Ji-Soo Kim

**Affiliations:** 1 Biomedical Research Institute, Seoul National University Bundang Hospital, Seongnam, Republic of Korea; 2 Department of Health Policy and Management, College of Medicine, Seoul National University, Seoul, Republic of Korea; 3 Department of Family Medicine, Seoul National University Bundang Hospital, Seongnam, Republic of Korea; 4 Department of Family Medicine, College of Medicine, Seoul National University, Seoul, Republic of Korea; 5 Department of Neurology, College of Medicine, Seoul National University, Seoul, Republic of Korea; 6 Department of Neurology, Dizziness Center, Clinical Neuroscience Center, Seoul National University Bundang Hospital, Seongnam, Republic of Korea

**Keywords:** diplopia, healthcare costs, healthcare utilization, ocular motor cranial nerve palsy, strabismus

## Abstract

Diplopia is a disabling condition with diverse etiologies, but population-based studies on its healthcare utilization and healthcare costs are limited in South Korea. We conducted a nationwide cross-sectional study using all claims data submitted to the Korean Health Insurance Review and Assessment Service in 2023. Patients with diplopia were identified using diagnostic codes for diplopia and strabismus. Direct healthcare costs, healthcare utilization patterns, and demographic characteristics were analyzed, with subgroup analyses for ocular motor cranial nerve palsies. Among 51.4 million insured individuals in South Korea, 89,447 patients with diplopia were identified, corresponding to a one-year prevalence of 0.17%. Age-specific prevalence revealed a bimodal age distribution. A total of 248,431 diplopia-related healthcare visits were recorded, primarily managed in ophthalmology (70.2%) and neurology (21.3%). The annual direct healthcare cost attributable to diplopia was ₩59.9 billion, and the mean cost per visit or admission day was ₩241,077, approximately 3.1 times the national average insurance benefit cost per outpatient visit or inpatient day (₩76,553). This nationwide claims-based study demonstrates that diplopia is associated with substantially higher healthcare costs than the national average across all medical conditions, with marked heterogeneity by age and underlying etiology. These findings underscore the need for age- and etiology-based, multidisciplinary strategies for optimized care and reducing healthcare costs of diplopia.

## Introduction

Diplopia, commonly referred to as double vision, is a condition that significantly impairs visual function and quality of life (1). It can be caused by a variety of underlying factors, including congenital abnormalities, ophthalmologic disorders, neurological deficits, and muscular dysfunction (2, 3). Accordingly, management of diplopia varies substantially depending on its etiology, often requiring involvement of multiple medical specialties and healthcare resources (4). As a result, the healthcare burden associated with diplopia is broad and complex, encompassing diverse medical services and treatment modalities.

Previous studies have predominantly focused on the clinical characteristics, etiological distribution, and management of diplopia, with particular emphasis on underlying causes and diagnostic approaches (3, 5–8). Despite its clinical importance, the healthcare utilization patterns and healthcare costs associated with diplopia have not been systematically investigated. A comprehensive evaluation of the healthcare burden related to diplopia may therefore provide important evidence to inform healthcare policy and resource allocation.

South Korea became a super-aged society in 2024, with more than 20% of the population aged 65 years or older, and is aging more rapidly than any other OECD countries (9, 10). Because diplopia in older adults frequently arises from microvascular and other acquired neurologic conditions whose incidence increases with age (2, 5, 11–13), the demand for its diagnostic evaluation and management, as well as the associated healthcare expenditure, is expected to grow. Nevertheless, the nationwide healthcare utilization and costs of diplopia have not been systematically estimated, and such evidence is needed to inform reimbursement policy and the planning of neuro-ophthalmologic services.

This study aims to address this gap by utilizing public claims data from the Health Insurance Review and Assessment Service (HIRA) of South Korea to assess the healthcare costs associated with diplopia and strabismus. Using the data from approximately 90,000 patients diagnosed with diplopia or strabismus in 2023, we evaluated the distribution of healthcare expenditures according to types of medical services and demographic characteristics of the patients. Through these analyses, we aimed to quantify the financial burden imposed on patients and the healthcare system, thereby providing evidence to inform cost-effective management strategies for diplopia.

## Methods

### Study design, setting, and population

This retrospective cross-sectional study analyzed nationwide healthcare utilization data and costs among patients with diplopia in South Korea. We used all claims data submitted to the HIRA between January 1 and December 31, 2023. South Korea operates a mandatory national health insurance system covering approximately 97% of the population, with the remaining individuals supported by Medical Aid (14). HIRA manages both National Health Insurance and Medical Aid data. Thus, HIRA claims data comprehensively capture healthcare utilization at the national level.

Patients with diplopia were identified using the 7th edition of the Korean Standard Classification of Diseases, the Korean version of the 10th edition of the International Standard Classification of Diseases. Diplopia-related conditions were defined by the presence of at least one of the following diagnostic codes: diplopia (H53.2), paralytic strabismus (H49), other strabismus (H50), and other disorders of binocular movement (H51). Because oculomotor (H49.0), trochlear (H49.1), and abducens (H49.2) nerve palsies are subcategories of paralytic strabismus (H49), which was one of our inclusion codes, patients with an isolated ocular motor cranial nerve palsy were captured even when no separate diplopia code was recorded. All individuals with at least one claim containing these diagnostic codes in the primary or an additional diagnosis field during the study period were included, and the total reimbursed cost of each such claim was attributed to diplopia. In addition, a subgroup analysis was performed for diplopia attributable to ocular motor cranial nerve palsies. This subgroup was identified using the specific diagnostic subcodes of paralytic strabismus (H49), including oculomotor nerve palsy (H49.0), trochlear nerve palsy (H49.1), and abducens nerve palsy (H49.2).

The primary endpoint of the study was the direct healthcare cost attributable to diplopia. Secondary endpoints included patterns of healthcare utilization such as outpatient visits, hospitalizations, and emergency department visits as well as demographic characteristics (age and sex), distributions by medical specialty, type of healthcare institution (tertiary referral hospitals, general hospitals, hospitals, and clinics), and geographic characteristics based on the location of healthcare institutions.

### Variables

Patient age and sex were obtained from the claims data. Comorbidity burden was assessed using the Charlson Comorbidity Index (CCI), calculated based on diagnostic codes recorded in the claims data (15). Hospitalization was defined as at least one inpatient admission during the study year. Healthcare utilization was evaluated based on the occurrence of outpatient visits, hospitalizations, and emergency department visits, and healthcare costs were calculated according to each type of healthcare utilization.

Information on healthcare institutions was obtained from the claims data to classify the type of healthcare institution and its geographic location. Geographic regions were originally categorized into 17 administrative areas and were further regrouped into the Seoul Capital Area (Seoul and Gyeonggi Province), six metropolitan cities (Incheon, Busan, Daegu, Gwangju, Daejeon, and Ulsan), and other regions.

### Statistical analyses

Baseline characteristics were summarized as means with standard deviations for continuous variables and as numbers with percentages for categorical variables. When individuals had multiple healthcare visits, each visit was analyzed as a separate case.

Total healthcare costs were presented as means with 95% confidence intervals, and mean costs per case were calculated by dividing the total healthcare costs by the number of diplopia-related visits. Age-specific prevalence rates were calculated by dividing the number of patients with diplopia in each age group by the corresponding general population of that age group, obtained from Statistics Korea, and were expressed per 100,000 population. No adjustment for sex or other covariates was applied. All statistical analyses were performed using SAS Enterprise Guide version 7.15 (SAS Institute, Cary, NC, U. S.), and a two-tailed *p* value < 0.05 was considered statistically significant.

### Ethics statement

This study was reviewed and approved by the Institutional Review Board of Seoul National University Bundang Hospital (X-2507-986-902). No informed consent was required from patients due to the nature of public data from HIRA.

## Results

### Demographic characteristics

**Figure 1:**
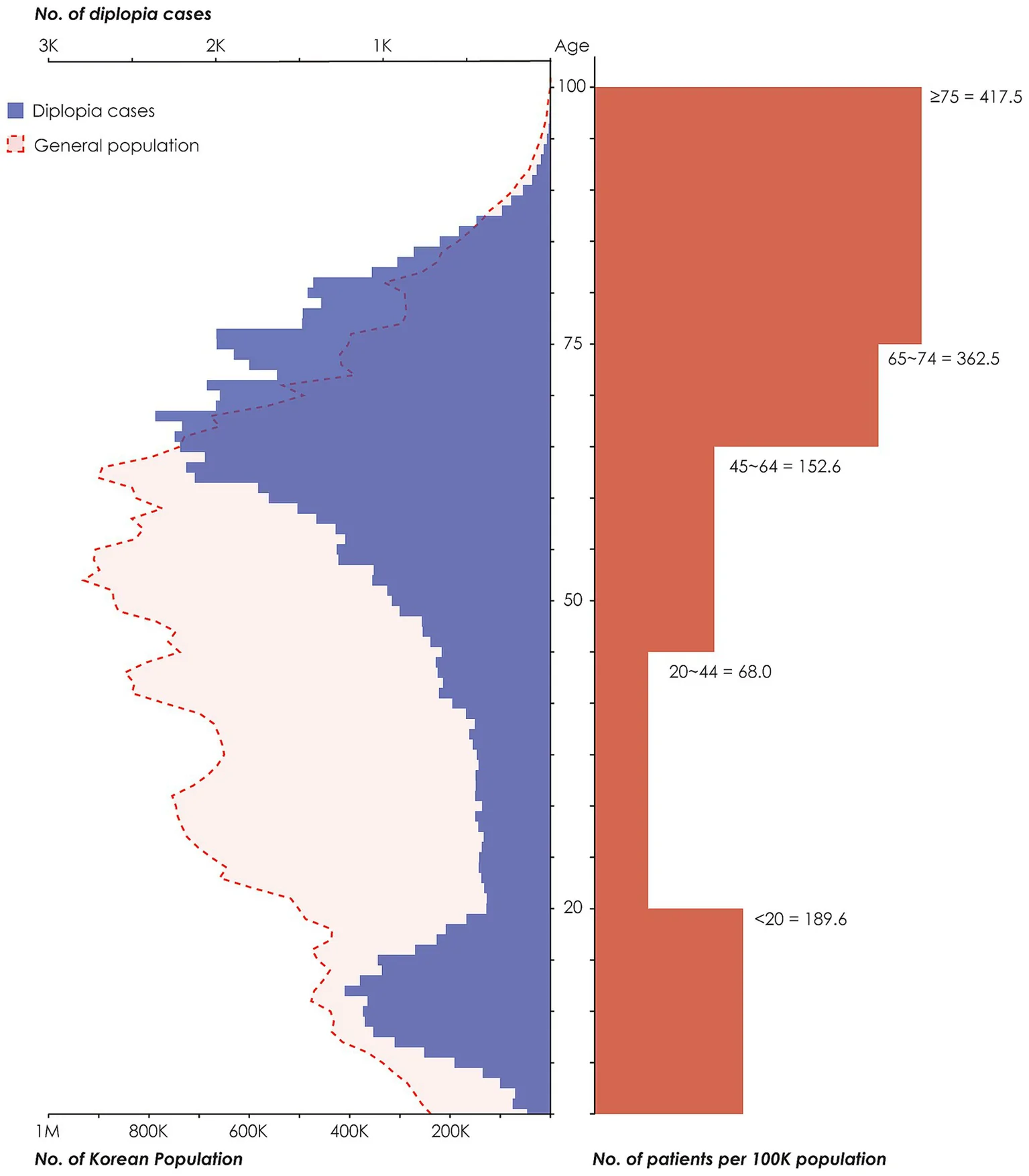
Distribution of age in general population (red dashed line) and patients with diplopia (blue bars) is shown in the left panel. Diplopia cases exhibited a bimodal age distribution, with an early peak in younger individuals and a second more prominent peak in older age groups. The right panel illustrates age-specific prevalence rates of diplopia per 100,000 population across predefined age categories.

In 2023, 89,447 patients were identified as having diplopia among 51,411,696 individuals covered by the national health insurance system in South Korea (16), corresponding to an overall prevalence of 0.17%. The baseline demographic and clinical characteristics of the total population and patients with diplopia are summarized in Table 1. Compared with the general population, patients with diplopia were significantly older, with a higher proportion of individuals aged 65 years or older, and showed a modest female predominance (Table 1). Age-specific prevalence of diplopia demonstrated a bimodal age distribution, with elevated rates at both extremes of age: 189 per 100,000 among individuals aged <20 years and 418 per 100,000 among those aged ≥75 years, compared with 68 per 100,000 in individuals aged 20–44 years (Figure 1).

**Table 1:** Overall characteristics of the study populations.

Characteristic	*N* (%)		
	Total population (*N* = 51,774,521)	Patients with diplopia (*N* = 89,447)	*p*-value
Age (Mean ± SD)	44.1 ± 21.5	53.1 ± 23.9	<0.001
<20	7,917,765 (15.29)	14,934 (16.70)	<0.001
20–44	17,470,542 (33.74)	11,884 (13.29)	
45–64	16,777,233 (32.40)	25,600 (28.62)	
65–74	5,620,207 (10.86)	20,374 (22.78)	
≥75	3,988,774 (7.70)	16,655 (18.62)	
Sex			
M	25,903,852 (50.03)	42,576 (47.60)	<0.001
F	25,870,669 (49.97)	46,871 (52.40)	
CCI			
0		33,670 (37.64)	
1		25,563 (28.58)	
≥2		30,214 (33.78)	

Data for the total population were obtained from Statistics Korea, whereas data for patients with diplopia were derived from the HIRA claims database. Values are presented as number (%) or mean ± standard deviation. CCI = Charlson comorbidity index.

### Patterns of healthcare utilization

A total of 248,431 hospital visits attributable to diplopia were identified in 2023 (Table 2). Among these visits, 3.7% resulted in hospital admissions and 3.0% involved emergency department visits. Most diplopia-related visits were managed in ophthalmology (70.2%), followed by neurology (21.3%), with the majority of visits concentrated in these two specialties. More than half of visits occurred at general hospitals or tertiary referral hospitals (58.8%), and most visits occurred at medical institutions located in the Seoul capital area and the six metropolitan cities (79.0%).

**Table 2:** Characteristics of study population by disease.

Characteristic	Diplopia (248,431)	Oculomotor nerve palsy (17,069)	Trochlear nerve palsy (22,593)	Abducens nerve palsy (23,863)
Age (Mean ± SD)				
<20	32,372 (13.03%)	504 (2.95%)	4,870 (21.56%)	923 (3.87%)
20–44	30,154 (12.14%)	1,370 (8.03%)	2,068 (9.15%)	2,576 (10.79%)
45–64	72,736 (29.28%)	5,333 (31.24%)	6,810 (30.14%)	7,612 (31.90%)
65–74	62,101 (25.00%)	4,861 (28.48%)	5,459 (24.16%)	6,856 (28.73%)
≥75	51,068 (20.56%)	5,001 (29.30%)	3,386 (14.99%)	5,896 (24.71%)
Sex				
M	126,084 (50.75%)	9,514 (55.74%)	14,167 (62.71%)	13,282 (55.66%)
F	122,347 (49.25%)	7,555 (44.26%)	8,426 (37.29%)	10,581 (44.34%)
CCI				
0	82,931 (33.38%)	3,263 (19.12%)	8,826 (39.07%)	5,606 (23.49%)
1	71,083 (28.61%)	5,021 (29.42%)	6,414 (28.39%)	6,817 (28.57%)
≥2	94,417 (38.01%)	8,785 (51.47%)	7,353 (32.55%)	11,440 (47.94%)
Admissions	8,986 (3.62%)	973 (5.70%)	966 (4.28%)	1,078 (4.52%)
Emergency room visits	3,751 (2.98%)	632 (3.70%)	233 (1.03%)	462 (1.94%)
Specialties				
Ophthalmology	174,340 (70.18%)	6,801 (39.84%)	16,077 (71.16%)	13,622 (57.08%)
Neurology	52,797 (21.25%)	6,915 (40.51%)	5,083 (22.50%)	7,666 (32.13%)
Neurosurgery	6,994 (2.82%)	1,584 (9.28%)	355 (1.57%)	926 (3.88%)
Internal medicine	5,425 (2.18%)	848 (4.97%)	358 (1.58%)	928 (3.89%)
Emergency medicine	2,485 (1.00%)	190 (1.11%)	125 (0.55%)	187 (0.78%)
Other departments	6,390 (2.57%)	731 (4.28%)	595 (2.63%)	534 (2.24%)
Type of medical institution				
Tertiary referral hospital	92,055 (37.05%)	7,561 (44.30%)	11,787 (52.17%)	10,791 (45.22%)
General hospital	53,913 (21.70%)	5,855 (34.30%)	5,653 (25.02%)	4,836 (28.65%)
Hospital	15,781 (6.35%)	789 (4.62%)	1,780 (7.88%)	1,554 (6.51%)
Clinic	86,682 (34.89%)	2,864 (16.78%)	3,373 (14.93%)	4,682 (19.62%)
Region				
Seoul Capital Area	129,388 (52.08%)	8,335 (48.83%)	12,013 (53.17%)	11,258 (47.18%)
Metropolitan cities	66,784 (26.88%)	4,661 (27.31%)	7,085 (31.36%)	7,274 (30.48%)
Other regions	52,259 (21.04%)	4,073 (23.86%)	3,495 (15.47%)	5,331 (22.34%)

### Healthcare costs

In 2023, the annual insurance benefit expenditure amounted to ₩92.3369 trillion (approximately USD 70.0 billion), corresponding to an average annual insurance benefit cost per capita of ₩1,796,030 (approximately USD 1,361.7). In addition, the average insurance benefit cost per outpatient visit or inpatient day was ₩76,553 (approximately USD 58.0) (16). The annual healthcare costs for diplopia amounted to ₩59.9 billion (approximately USD 45.4 million) in 2023 (Table 3). The mean direct healthcare cost per diplopia-related hospital visit or admission day was ₩241,077 (approximately USD 182.8, 95% CI, ₩236,977 – ₩245,177), which was approximately 3.1 times the national average insurance benefit cost per outpatient visit or inpatient day across all conditions (₩76,553, approximately USD 58.0) (16).

**Table 3:** Direct healthcare costs of diplopia and ocular motor cranial nerve palsies.

Cost measure	Diplopia	Oculomotor nerve palsy	Trochlear nerve palsy	Abducens nerve palsy
No. of hospital visits	248,431	17,069	22,593	23,863
Annual healthcare costs, ₩	59,891,067,263	6,978,693,422	4,873,751,341	7,069,062,725
Mean direct healthcare cost per visit or inpatient day, ₩	241,077	408,852	215,720	296,235
95% CI	236,977–245,177	377,627–440,077	207,048–224,391	277,019–315,451

### Subgroup analyses according to ocular motor cranial nerve palsies

Among diplopia-related hospital visits, ocular motor cranial nerve palsies constituted a substantial subgroup, comprising 17,069 (6.9%) visits for oculomotor nerve palsy, 22,593 (9.1%) for trochlear nerve palsy, and 23,863 (9.6%) for abducens nerve palsy (Table 2).

**Figure 2:**
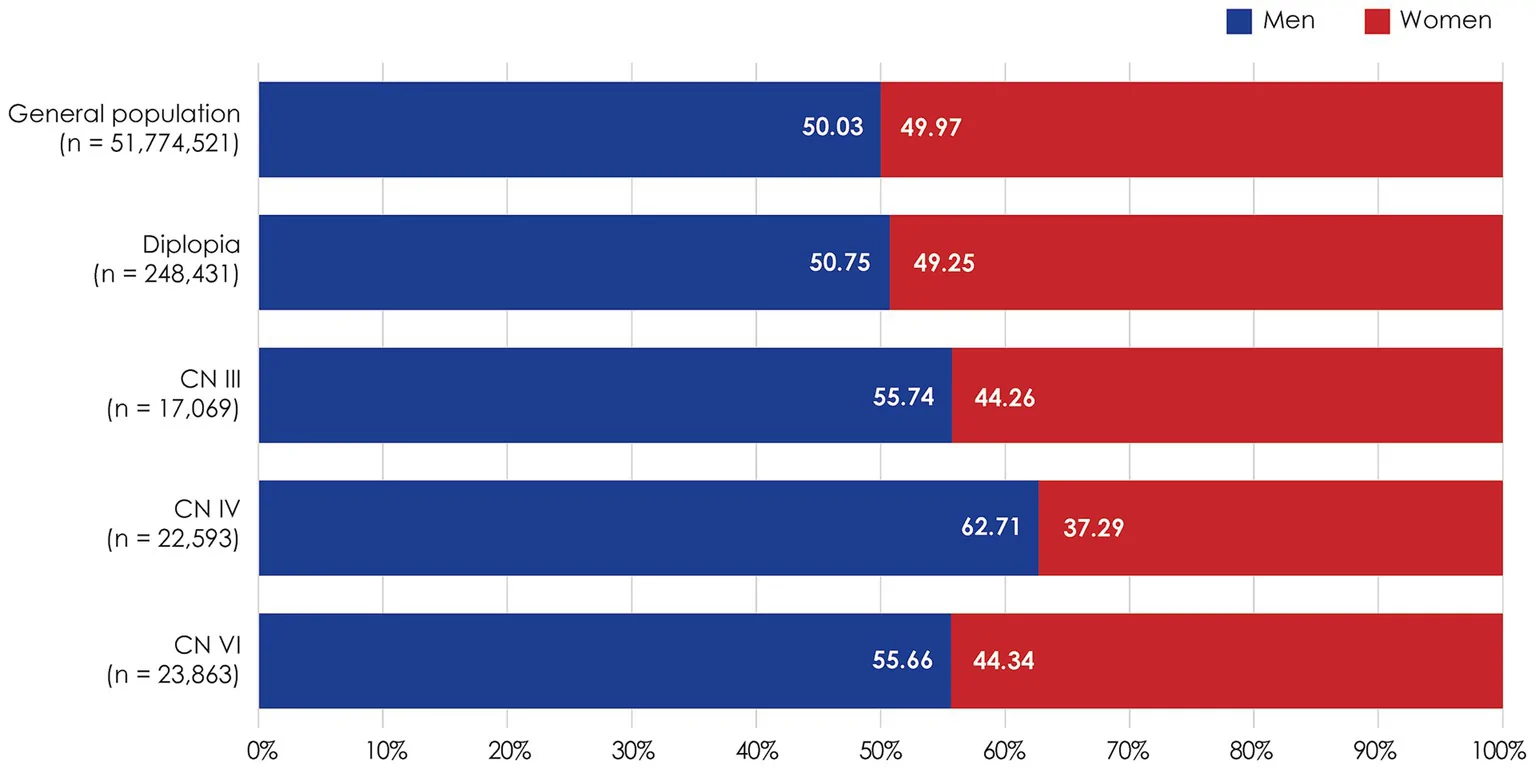
The distribution of sex is shown for the general population, overall diplopia, and ocular motor cranial nerve palsies. CN III: oculomotor nerve palsy, CN IV: trochlear nerve palsy, CN VI: abducens nerve palsy.

The age distribution differed markedly across cranial nerve palsy subtypes. Trochlear nerve palsy demonstrated a younger age profile, with 21.6% of visits occurring in individuals younger than 20 years, whereas oculomotor and abducens nerve palsies were predominantly observed in middle-aged and older adults. In particular, individuals aged 45–74 years accounted for the largest proportion of visits in both oculomotor and abducens nerve palsies. A male predominance was observed across all ocular motor cranial nerve palsies, in contrast to the nearly balanced sex distribution seen in the overall diplopia population (Figure 2). This pattern was most pronounced in trochlear nerve palsy, in which males accounted for 62.7%, followed by oculomotor nerve palsy (55.7%) and abducens (55.7%).

Comorbidity profiles also varied by cranial nerve subtype. Patients with oculomotor and abducens nerve palsies exhibited a higher comorbidity burden, with 51.5 and 47.9% of visits, respectively, occurring in individuals with a CCI score of ≥ 2. In contrast, trochlear nerve palsy was more frequently observed in patients without significant comorbidities, with 39.1% of visits occurring in those with a CCI score of 0.

Hospital admissions and emergency department visits were most frequent among patients with oculomotor nerve palsy (5.7 and 3.7%, respectively). In contrast, trochlear and abducens nerve palsies were associated with lower rates of hospitalization (4.3–4.5%) and emergency department utilization (1.0–1.9%). Trochlear nerve palsy was predominantly managed by ophthalmology (71.2%), whereas neurology accounted for a substantial proportion of visits for oculomotor (40.5%) and abducens nerve palsies (32.1%). The majority of visits for ocular motor cranial nerve palsies were managed at tertiary referral or general hospitals, accounting for more than 70% of visits across all three subtypes. This proportion was highest for oculomotor nerve palsy (78.6%), followed by trochlear (77.2%) and abducens nerve palsy (72.1%). Clinic-based care was less common for cranial nerve palsies than for diplopia overall. Across all ocular motor cranial nerve palsy subtypes, visits were predominantly concentrated in the Seoul capital area and metropolitan cities, exceeding three-quarters of visits in each subtype.

Among ocular motor cranial nerve palsies, the total annual direct healthcare costs were ₩7.0 billion (approximately USD 5.3 million) for oculomotor nerve palsy, ₩4.9 billion (approximately USD 3.7 million) for trochlear nerve palsy, and ₩7.1 billion (approximately USD 5.4 million) for abducens nerve palsy (Table 3). The mean direct healthcare cost per visit or inpatient day was highest for oculomotor nerve palsy (₩408,852, approximately USD 310.0, 95% CI, ₩377,627 – ₩440,077), followed by abducens nerve palsy (₩296,235, approximately USD 224.6, 95% CI, ₩277,019 – ₩315,451) and trochlear nerve palsy (₩215,720, approximately USD 163.5, 95% CI, ₩207,048 – ₩224,391).

## Discussion

Diplopia imposes a healthcare burden that is disproportionate to its common perception as a minor symptom, and this burden is particularly pronounced in South Korea, which is undergoing the most rapid population aging among OECD countries. In this claims-based analysis, we quantified the healthcare utilization and direct healthcare costs associated with diplopia in South Korea. Using data from nearly 90,000 patients in 2023, we found that diplopia accounted for a substantial healthcare burden, with a mean cost per outpatient clinic visit or inpatient day approximately threefold higher than the national average across all medical conditions. Although patients with diplopia were predominantly older adults, age-specific prevalence revealed a bimodal age distribution, with disproportionately higher visit prevalence among individuals younger than 20 years and those aged 75 years or older. Most diplopia-related visits were managed by ophthalmologists and neurologists, and more than half occurred at general or tertiary referral hospitals, reflecting the need for specialized diagnostic evaluation. According to subgroup analyses, ocular motor cranial nerve palsies were associated with higher comorbidity burdens, greater rates of hospitalization and emergency department use, and markedly higher per-visit costs compared with diplopia overall.

The present study demonstrates a bimodal age distribution of diplopia, with concurrently increased incidence observed in both pediatric/adolescent populations and in older adults (Figure 1). This pattern is further supported by the subgroup analysis of trochlear nerve palsy, in which individuals younger than 20 years accounted for more than 20% of patients, while those aged 65 years or older comprised nearly 40% of cases. Direct comparison with previous studies is challenging, as most existing research on diplopia has focused on either pediatric or adult populations, or on specific etiologic subgroups, rather than defining diplopia as a symptom and analyzing its incidence across the entire age spectrum in a population-based setting (4–7, 11–13). Nevertheless, studies investigating the epidemiologic characteristics of isolated trochlear nerve palsy have consistently reported a bimodal age distribution (5, 13). The similar age pattern observed in our study suggests that diplopia in individuals younger than 20 years is largely attributable to congenital etiologies, whereas diplopia in older adults, particularly those aged 60 years or older, is predominantly associated with microvascular etiologies, in line with previously reported age-dependent etiologic transitions (5, 13).

These age-dependent etiologic variations were further reflected in the healthcare expenditure patterns of ocular motor cranial nerve palsies. Trochlear nerve palsy, which predominantly affects younger populations, incurred lower mean direct healthcare costs per visit relative to overall diplopia and other cranial nerve palsies. This disparity is likely due to the predominance of congenital etiologies in younger patients, which generally present as long-standing conditions with a low likelihood of serious intracranial pathology and are therefore less likely to necessitate intensive diagnostic evaluation, often allowing for outpatient-based management (13). Notably, trochlear nerve palsy was associated with the lowest comorbidity burden (39.1% of visits in patients with a Charlson Comorbidity Index of 0) and the youngest age distribution among the ocular motor cranial nerve palsies. This pattern likely reflects the fact that a substantial proportion of fourth nerve palsies presenting in adulthood represent decompensated congenital palsies rather than newly acquired vascular or neurologic disease. Conversely, oculomotor and abducens nerve palsies—more common in older adults and often linked to microvascular or neurological pathologies—demonstrated significantly higher per-visit costs, reflecting an increased reliance on inpatient care, emergency services, and advanced neuroimaging. These findings underscore that the economic burden of diplopia is disproportionately distributed across etiologic subgroups, driven largely by age-specific pathologies and healthcare utilization trends.

In this study, most diplopia-related healthcare utilization occurred within ophthalmology and neurology, reflecting the dual ocular alignment and neurologic mechanisms underlying diplopia (3). Although age-stratified analyses by clinical specialty were not directly performed, ophthalmology utilization was higher for trochlear nerve palsy, which is more prevalent in younger individuals, whereas neurology utilization was more frequent for oculomotor and abducens nerve palsies, which are more commonly observed in older adults. These findings indirectly suggest that patterns of specialty care vary according to the underlying etiology of diplopia. In cases where diplopia raises concern for acquired neurological causes, more extensive diagnostic evaluation may be required (8). Accordingly, differences in specialty-specific patterns of care appear to align with observed variations in healthcare utilization and costs, underscoring the potential value of coordinated, cross-disciplinary approaches in the evaluation and management of diplopia. Notably, neurosurgery accounted for a higher proportion of visits among patients with oculomotor nerve palsy compared with diplopia overall and other ocular motor cranial nerve palsies. This finding may be explained by the relatively higher prevalence of neoplastic or vascular etiologies in oculomotor nerve palsy, as previously reported, which often necessitate neurosurgical consultation and management (7).

We found that more than half of diplopia-related healthcare utilization occurred at general or tertiary referral hospitals, with a substantial concentration in the Seoul capital area and major metropolitan cities. This pattern likely reflects the need for specialized diagnostic resources, including neuro-ophthalmologic expertise and advanced imaging, particularly in older patients or in those with suspected neurologic etiologies. The concentration of care in metropolitan regions further suggests that clinical expertise and infrastructure required for the evaluation of diplopia are disproportionately located in urban centers, consistent with documented regional disparities in healthcare resources in South Korea. Limited availability of specialized care in smaller cities is not unique to diplopia but represents a broader challenge observed across various medical conditions (17).

For international context, a prior U.S. study reported approximately 850,000 diplopia-related ambulatory and emergency department visits annually (18). A direct comparison of healthcare utilization or costs between the two countries was not attempted, as it would be confounded by differences in population standardization, diagnostic coding, underlying disease prevalence, and cost definitions. Notably, South Korea is characterized by high healthcare accessibility, with overall healthcare utilization among the highest across OECD nations (19).

Several limitations of this study should be acknowledged. First, this analysis was based on claims data, which lack detailed clinical information such as symptom severity, examination findings, imaging results, and exact etiologic confirmation. Consequently, etiologic classifications relied on diagnostic codes, and while ocular motor cranial nerve palsies accounted for approximately one-quarter of diplopia-related visits, the precise distribution of the remaining heterogeneous causes (e.g., comitant strabismus, thyroid eye disease, myasthenia gravis, or central causes) could not be fully resolved. Second, healthcare utilization and cost estimates reflect reimbursed services captured in claims data and may not fully represent indirect costs or non-reimbursed care. Moreover, because costs were attributed to any claim carrying a diplopia-related code, encounters in which diplopia reflected a resource-intensive underlying condition may have inflated the costs estimated as attributable to diplopia. Third, although associations among age, etiology, healthcare utilization, and costs were observed, causal relationships cannot be inferred due to the observational nature of the study. Finally, healthcare costs were not analyzed according to the treating specialty; whether the cost of managing a given ocular motor cranial nerve palsy differed depending on whether the patient was cared for by an ophthalmologist or a neurologist could not be determined in this study, and we plan to examine this aspect in closer detail in future research.

In conclusion, this nationwide claims-based study demonstrates that diplopia is associated with healthcare costs approximately threefold higher than the national average in South Korea, highlighting its considerable burden on the healthcare system. These findings further indicate marked heterogeneity in healthcare utilization and costs according to age and underlying etiology, suggesting that diplopia should not be regarded as a uniform clinical entity but rather as a condition that warrants age- and etiology-informed, multidisciplinary approaches. Our results may serve as a foundation for discussions on healthcare system planning and policy development aimed at addressing the burden of diplopia.

## Data Availability

The data analyzed in this study is subject to the following licenses/restrictions: the data were obtained from the Health Insurance Review and Assessment Service (HIRA) under a data use agreement and are not publicly available. Access to the data is subject to HIRA regulations and requires approval from the Health Insurance Review and Assessment Service. Requests to access these datasets should be directed to jisookim@snu.ac.kr.
